# Formulation of an aloe-based product according to Iranian traditional medicine and development of its analysis method

**DOI:** 10.1186/s40199-017-0185-x

**Published:** 2017-08-29

**Authors:** Elham Moein, Homa Hajimehdipoor, Tayebeh Toliyat, Rasool Choopani, Maryam Hamzeloo-Moghadam

**Affiliations:** 1 Department of Traditional Pharmacy, School of Traditional Medicine, Shahid Beheshti University of Medical Sciences, Tehran, Iran; 2 Traditional Medicine and Materia Medica Research Center and Department of Traditional Pharmacy, School of Traditional Medicine, Shahid Beheshti University of Medical Sciences, No. 8 Shams Alley, Vali-e-Asr Street, Tehran, 1516745811 Iran; 3 Department of Pharmaceutics, Faculty of Pharmacy, Tehran University of Medical Sciences, Tehran, Iran; 4 Department of Traditional Medicine, School of Traditional Medicine, Shahid Beheshti University of Medical Sciences, Tehran, Iran

**Keywords:** Aloe, *Ayarij-e-Faiqra*, HPLC, Formulation, Quality Control, Iranian traditional medicine

## Abstract

**Background:**

Currently, people are more interested to traditional medicine. The traditional formulations should be converted to modern drug delivery systems to be more acceptable for the patients. In the present investigation, a poly herbal medicine “*Ayarij-e-Faiqra*” (AF) based on Iranian traditional medicine (ITM) has been formulated and its quality control parameters have been developed.

**Methods:**

The main ingredients of AF including barks of *Cinnamomum zeylanicum* Blume and *Cinnamomum cassia* J. Presl, the rhizomes of *Nardostachys jatamansi* DC., the fruits of *Piper cubeba* L.f., the flowers of *Rosa damascena* Herrm., the oleo gum resin of *Pistacia terebinthus* L. and *Aloe* spp. dried juice were powdered and used for preparing seven tablet formulations of the herbal mixture. Flowability of the different formulated powders was examined and the best formulations were selected (F6&F7). The tablets were prepared from the selected formulations compared according to the physical characteristics and finally, F7 was selected and coated. Physicochemical characters of core and coated AF tablets were determined and the HPLC method for quantitation of aloin as a marker of tablets was selected and verified according to selectivity, linearity, precision, recovery, LOD and LOQ.

**Results:**

The results showed that core and coated AF tablets were in agreement with USP requirements for herbal drugs. They had acceptable appearance, disintegration time, friability, hardness, dissolution behavior, weight variation and content uniformity. The amount of aloin in tablets was found 123.1 mg/tab. The HPLC method for aloin determination in AF tablets was verified according to selectivity, linearity (5–500 μg/ml, r^2^:0.9999), precision (RSD: 1.62%), recovery (108.0%), LOD & LOQ (0.0053 & 0.0161 μg/ml).

**Conclusions:**

The formulated tablets could be a good substitute for powder and capsules of AF in ITM clinics with a feasible and precise method for its quality control.

**Graphical abstract:**

Ayarij-e-Faiqra formulation
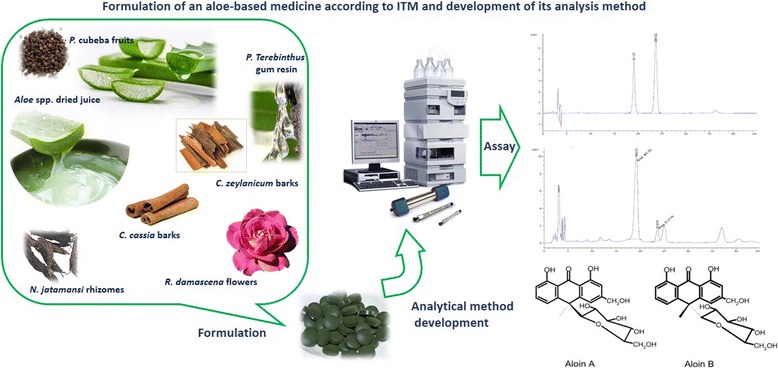

## Background

The world is moving towards traditional and complementary medicine to increase efficacy, safety and cost-effectiveness of treatments [1]. The World Health Organization (WHO) has admitted the influence of traditional and complementary medicine in the well-being of people and has developed instructions for supporting the countries in establishing their national policies about traditional medicine [2].

Iranian traditional medicine (ITM), its summit by Avicenna in tenth century, is one of the oldest and richest holistic medical systems that has promote healthy life [1]. ITM believes that balance in the four humors (phlegm, blood, yellow bile and black bile) establishes a healthy body and imbalance would result in illness. A healthy human body expels the waste humors through urine, feces, perspiration, sputum, menses (in women) and some other body’s secretions. Any obstruction and occlusion can cause retention of waste humors. ITM believes in regulation or elimination of the waste humor to cure diseases [3]. Purgation is one of the simplest and most effective ways to clear waste humors from the body. Purgative remedies not only used for treatment of constipation but also for expelling the waste humors from vessels, membranes and every parts of the body (as a systemic agent) [4]. *“Ayarij”* is a multi-ingredient purgative agent that has been used for treatment of many diseases. *“Ayarij-e-Faiqra”* (AF), is the most famous kind of *“Ayarij”*, with the main ingredient being *Aloe* spp. dried juice [4–6]. It has several applications in ITM, for example it is used in form of gargle for tremor relief [7], or in oral dosage forms as cleaning agent for removing death tissue in gastric ulcer [8, 9] and treatment of obesity [10]. AF is one of the most important multi herbal formulations in ITM first described by Hippocrates as a purgative agent [11]. In order to increase its efficacy and decrease side effects especially abdominal cramps, several other plants have been added to aloe in the formulation [4–6, 11, 12]. Different formulation of AF were found in ITM textbooks and a variety of plants have been suggested in different formulations of AF [4–6]; among them, a prescription containing *Cinnamomum zeylanicum* Blume*, Cinnamomum cassia* J.Presl, *Nardostachys jatamansi* DC., *Piper cubeba* L.f., *Rosa damascene* Herrm., *Pistacia terebinthus* L. and *Aloe* spp. as the main components, found more than others in ITM textbooks. In Table 1, the scientific name, family, common and traditional names, temperament and the used parts of plants in AF formula have been summarized. Mixture of the powdered mentioned species has been filled in hard gelatin capsules (500 mg each) and used in ITM clinics; its effectiveness has been established during many years.

**Table 1:** The ingredients of “*Ayarij-e-Faiqra*”

No.	Scientific name	Family	Common name	Traditional name	Temperament	Part used
1	*Aloe* spp.	Liliaceae	Bitter aloe	Sabr	Hot and dry	Dried juice
2	*Cinnamomum zeylanicum*	Lauraceae	Cinnamon	Darsini	Hot and dry	Bark
3	*Cinnamomum cassia*	Lauraceae	Cassia, Chinese cinnamon	Salikhah	Hot and dry	Bark
4	*Nardostachys jatamansi*	Valerianaceae	Indian valerian, spikenard	Sumbul-uttib	Hot and dry	Rhizome
5	*Pistacia terebinthus*	Anacardiaceae	Saghez	Elkol-botm	Hot and dry	Oleo gum resin
6	*Piper cubeba*	Piperaceae	Cubebs	Kababah	Hot and dry	Fruit
7	*Rosa damascena*	Rosaceae	Damask rose	Ward	Cold and dry	Flower

*Aloe* spp. dried juice, the main component of AF, has been traditionally used in small doses as a tonic and for improvement of digestion; while in larger doses, it has been used as a laxative, purgative and emmenagogue [13]. The high content of anthraquinones and anthrones in *Aloe* spp. dried juice stimulate the intestinal motility and increase passage through the digestive system [14]. Thus, its effectiveness may be largely due to differing levels of aloe emodin and/or aloin present in the dried juice; therefore, it is essential to use a standardized dosage form to access a suitable and repeatable therapeutic response [15, 16].

Based on the WHO guidelines, the active pharmaceutical constituents of herbal recipes should be analyzed by standard methods before accomplishing clinical trials. Defining values for examining the quality of natural products is not easy because they usually possess a variety of complex constituents; therefore, modern analytical techniques are expected to help in circumventing this problem [2, 17]. In the present investigation, a film coated tablets of AF has been prepared and its quality control parameters have been developed.

## Methods

### Plant materials

All required herbs and *Aloe* spp. dried juice were purchased from local markets in Tehran, Iran. They were identified by the botanists of the Traditional Medicine and Materia Medica Research Center (TMRC), Shahid Beheshti University of Medical Sciences, Tehran, Iran and their voucher specimens were deposited at TMRC Herbarium for further reference (No. 342, 341, 343, 345, 346, 348 and 347 HMS, for the barks of *C. zeylanicum* and *C. cassia,* the rhizome of *N. jatamansi,* the fruit of *P. cubeba*, the flower of *R. damascena*, the oleo gum resin of *P. terebinthus* and dried juice of *Aloe* spp., respectively).

### Chemicals

Acetonitrile and Methanol (HPLC grade) were purchased from Duksan Company (Korea). Aloin standard material was prepared from ROTH (Karlsruhe, Germany) and other chemicals and solvents were provided from Merck (Germany). Deionized water was used in all experiments.

### Instrumentation

HPLC analysis was carried out on Agilent Technologies equipped with a vacuum degasser, auto-sampler and UV detector. The spectrophotometric detection was performed at 295 nm. ChemStation software was utilized for instrument control, data collection and data processing. The column was C_18_ (4.6 × 250 mm, 5 μm). The mobile phase was water: acetonitrile (80:20) for 50 min. The flow rate was 1 ml/min. The injection volume for all samples and standard solutions was 20 μl. The hardness of the tablets was determined using a hardness tester (Model TBH28, Erweka, Germany). Friability of tablets was assessed by Pharma-test friabilator (Model S. 48-3 cm, Iran). Disintegration time and dissolution behavior of tablets were determined using disintegration testers (Model ZT3, Erweka, Germany) and dissolution testers from Kavosh Co., Iran. The tablets were pressed with EKO model single-punch tablet machine (Erweka, Germany).

### Physicochemical analysis of crude herbs

Quality control assessments were performed for each herbal sample. Foreign matters, loss on drying, solubility, alcohol and water soluble extractives, total ash and acid insoluble ash were obtained and evaluated according to pharmacopeia. Aloin content for *Aloe* spp., total phenolic contents for *R. damascena* and essential oil content for other herbs were measured [18–20].

### Pre-formulation studies

*Aloe* spp. dried juice (6.5 parts), *C. zeylanicum* (1 part), *C. cassia* (1 part), *N. jatamansi* (1 part), *P. cubeba* (1 part) and *R. damascena* (1 part) were powdered, passed through mesh 80 sieve and mixed. Then, *P. terebinthus* oleo gum resin was added to this mixture (in ratio 1.5) and sieved several times to obtain a uniform mixture and passed from mesh 18 sieves. Seven formulations using various ingredients containing avicel PH 102, Corn starch, croscarmellose sodium, crospovidone, colloidal silicon dioxide and magnesium stearate were prepared. All components except for the lubricant were mixed by cubic mixer. At the final step, magnesium stearate was added. Then, the flowability of formulations was determined on the basis of Carr’s index, Hausner ratio and angle of repose [21, 22].

### Preparation of AF tablets

Regarding the pre-formulation studies, two best formulations (F6, F7) were prepared by dry granulation method [22]. Then physicochemical properties of F6 and F7 were evaluated. Considering the results, the more suitable formulation (F7) was selected [19]. To improve the appearance of the tablet, protect the components from degradation during storage and to cover the unpleasant taste, smell and color [22], the tablets were coated using a solution containing White Opadry (10 g), D & C Green color (20 mg) and purified water (q.s to 100 ml).

### Quality control of AF tablets

The prepared tablets underwent various physiochemical tests and pharmaceutical parameters, including appearance, diameter, thickness, weight variation, friability, disintegration time, hardness, assay of aloin, uniformity of dosage units, loss on drying and dissolution behavior according to USP [19].

#### Determination of aloin in tablets

Since *Aloe* spp. dried juice is the main ingredient and major constituent of AF; the content of aloin was assessed in tablets according to USP-38 monograph [19] for aloe with some modifications as follows:

#### Sample preparation

Twenty tablets were powdered and average of one tablet was weighed and suspended in 75 ml methanol. The mixture was placed in an ultrasonic bath for 30 min and then filtered. The filtrate was transferred to a 100 ml volumetric flask and adjusted with the solvent to volume. One ml of the solution was transferred to a 50 ml volumetric flask, and diluted with the solvent to volume. The obtained solution was filtered through a membrane filter (0.45 μm) prior to injection, and 20 μl of the final solution was injected into the HPLC system.

#### Standard preparation

Standard solution of aloin (0.02 mg/ml) was prepared in methanol: water (1:1).

Sample and standard solutions were injected to HPLC system tree times and content of aloin in the tablets was calculated using AUC of standard and samples peaks of aloin in the chromatograms.

#### Determination of content uniformity of AF tablets

Since the aloin content of the tablets was less than 25% weight of each tablet, content uniformity test was performed by using HPLC with the same method as the assay test [19].

#### Dissolution test of AF tablet

Dissolution test was performed on six tablets. The USP apparatus 2 (paddle) at a speed of 75 rpm, with 900 ml distilled water as the dissolution medium was used and the samples were analyzed after 60 min. The amount of dissolved aloin was determined by HPLC using 5 ml filtered portions of the samples and aloin standard solution (0.1 mg/ml) in methanol: water (1:1). (Q) more than 75% was considered as acceptable for dissolution test.

#### Stability assessment of AF tablets

Laboratory accelerated stability test was performed on AF coated tablets. Forty tablets were packed in a polyethylene container and kept at 40 ± 2 °C temperature and 75 ± 5% humidity for 30 days. Then physicochemical characteristics of the tablets were determined [23].

### Method verification for assay of aloin in AF tablets

The method which was originated from *Aloe* spp. monograph (USP-38) was verificated for assay of aloin in AF tablets [19]. The method verification was performed through selectivity, linearity, precision, recovery, LOD and LOQ [24–26].

#### Selectivity

Selectivity is defined as the capability of the technique for precise measurement of the analyte response in the presence of all interferences; therefore, placebo (AF without *Aloe* spp.) and the solvent chromatograms were examined and the aloin peak in sample chromatogram was evaluated for resolution from the nearest peak.

#### Linearity

The correlation between the concentration of aloin and the obtained absorbance was evaluated as linearity. The determination coefficient (r^2^) was measured by the least-square analysis. The calibration lines were achieved by three replicates of each concentration of aloin (5–500 μg/ml), to determine the consistency of the response.

#### Precision

Consistent response of a measurement under unchanged conditions is known as precision. Three real samples were analyzed according to assay method and the relative standard deviations (RSDs%) were measured. Each sample was evaluated by HPLC thrice.

#### Recovery

The nearness between what has been achieved in the experiment and the real value is defined as recovery. Recovery confirms that no missing or absorbance has happened during the process. Four samples were prepared according to the assay method. One part was used as the real sample and the others had been spiked with 50 mg of aloin standard material. Each sample was injected three times into HPLC.

#### LOD and LOQ

Limit of detection and limit of quantitation were assessed using 3.3σ/s and 10σ/s expressions respectively, where σ is the intercept standard deviation and s is the slope of calibration curve.

## Results

The results of physicochemical analysis of crude herbs have been reported in Table 2.

**Table 2:** Physicochemical analysis of crude herbs in AF formulation

Ingredients	Assay	Essential oil	Foreign matter	Total ash	Acid insoluble ash	Alcohol soluble extractive	Water soluble extractive	Loss on drying	Alcohol insoluble substances
*Aloe* spp.	58.4 ± 0.3% Aloin, (NLT 6%)^a^	-	-	1.1% (NMT 4%)	-	-	79.0% (NLT 50%)	8.7% (NMT 12%)	1.8% (NMT 10%)
*Cinnamomum zylanicum*	-	2.5% (NLT 1%)	0.6% (NMT 2%)	2.5% (NMT 3%)	0.2% (NMT 2%)	15.6% (NLT 2%)	20.4% (NLT 3%)	-	-
*Cinnamomum cassia*	-	2.3%^b^	0.7% (NMT 2%)	3.1% (NMT 7%)	0.4% (NMT 1%)	13.7% (NLT 4%)	19.8% (NLT 7%)	-	-
*Nardostachys jatamansi*	-	1.6% (NLT 0.1%)	0.9% (NMT 5%)	5.0% (NMT 9%)	1.9% (NMT 5%)	4.9% (NLT 2%)	10.7% (NLT 5%)	-	-
*Piper cubeba*	-	2.1%	1.3%	6.5%	0.8%	20.2%	17.6%	-	-
*Pistacia terebinthus*	-	5.8%	0.0%	0.2%	0.1%	-	-	-	-
*Rosa damascena*	4.0% total phenolics as pyrogallol	0.1%	0.1% (NMT 2%)	3.5% (NMT 7.5%)	0.9% (NMT 1%)	31.0% (NLT 15%)	46.0% (NLT 24%)	-	-

^a^The data in parenthesis are acceptable ranges in Pharmacopoeia (USP/Unanian Pharmacopoeia)

^b^There is no acceptable range for some data

### Pre-formulation studies

Different formulations of AF tablets have been presented in Table 3.

**Table 3:** Ingredients of AF tablets in pre-formulation studies

Ingredients	Formulations							Function
	F1	F2	F3	F4	F5	F6	F7	
Herbal powder (mg)	500	500	500	500	500	500	500	Active ingredient
Microcrystalline cellulose (Avicel PH 102) (mg)	170	165	105	65	-	-	-	Diluent, disintegrant
Corn starch (mg)	-	-	65	105	170	195	190	Diluent, binder, disintegrant
Croscarmellose sodium (mg)	-	-	-	10	-	-	-	Disintegrant
Crospovidone (mg)	20	20	15	5	15	-	-	Disintegrant
Colloidal silicon dioxide (mg)	-	5	5	5	5	-	-	Glidant
Magnesium stearate (mg)	10	10	10	10	10	5	10	Lubricant
Total (mg)	700	700	700	700	700	700	700	-
*Angle of repose (* ^*°*^ *)*	34	29	28	25	25	24	21	-
*Carr’s index (%)*	18.18	18.64	15.52	16.36	15.09	14.29	12.7	-
*Hausner ratio*	1.22	1.23	1.18	1.19	1.17	1.16	1.14	-

The angle of repose revealed that the flowability of the F2-F7 formula were “good”, while it was “acceptable” for F1 formula. Flowability of the powder formulations was in the following order: F7 > F6 > F4 = F5 > F3 > F2 > F1. The Carr’s index and Hausner ratio are indirect methods for predicting the powder flow characteristics. The results of the Carr’s index of F6 and F7 formula were excellent; F3, F4 and F5 were good and F1, F2 formula fair to passable. Arrangement of flowability was F7 > F6 > F5 > F3 > F4 > F1 > F2. All formulations showed “good” Hausner ratio (<1.25) in accordance with the flowability arrangement of the Carr’s index. According to the chart of relationship between angle of repose and the Carr’s index, F7 and F6 formula showed better flowability compared to the other formula. Table 4 has demonstrated physical properties of AF tablets prepared according F6 and F7 formula.

**Table 4:** Physical properties of F6 and F7 formulations

Tests	AF formula	
	F6	F7
Appearance	brown with white spots, smooth and biconvex	brown with white spots, smooth and biconvex
Diameter (mm)	13.00 ± 0.01	13.00 ± 0.01
Thickness (mm)	5.20 ± 0.10	5.20 ± 0.05
Weight variation (mg)	697.10 ± 18.10	698.30 ± 17.55
Friability (%)	0.35	0.29
Hardness (N)	66.0 ± 9.0	66.5 ± 7.5
Disintegration time (min)	Max: 9:15 Min: 6:00	Max: 7:00 Min: 5:35

As it is obvious in Table 4, the thickness and diameter of tablets were not significantly different in formulations. The results have shown that F7, F6 had a narrow weight variation range. Both formulations showed appropriate hardness (60–100 N) and friability (less than 1%). F7 formula showed better physical properties with appropriate flowability, Carr’s index and Hausner ratio; thus F7 which contained 500 mg of herbal mixture, 190 mg of corn starch and 10 mg of magnesium stearate per tablet was considered as the suitable formula for coating.

### Determination of physicochemical characteristics of core and coated AF tablets

**Fig. 1:**
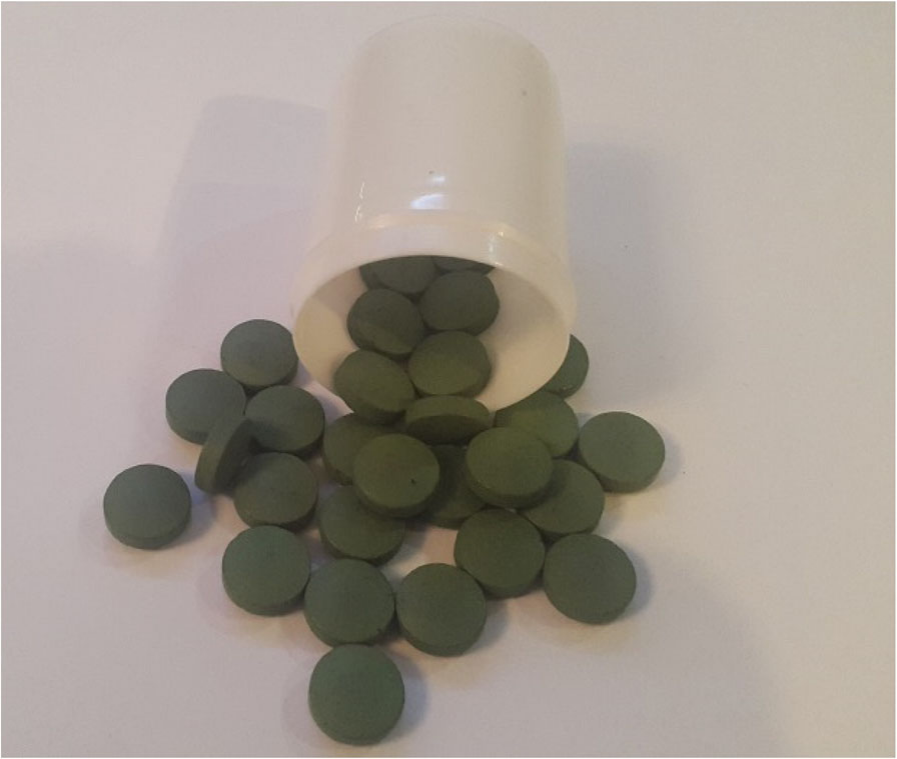
*“Ayarij-e-Faiqra”* film coated tablets

All physicochemical characteristics of AF tablets (core and coated) have been summarized in Table 5. AF coated tablets have been shown in Fig. 1.

**Table 5:** Physicochemical characteristics of AF tablets

Tests	Results		
	Core	Coated tablets	Coated tablets after 30 days
appearance	Round, biconvex, dark-brown tables with white spots	Round, biconvex, green tablets	Round, biconvex, green tablets
Thickness	5.2 mm	5.8 mm	5.8 mm
Diameter	13.0 mm	13.3 mm	13.3 mm
Wight variation	680.7–715.8 mg Mean: 698.4 mg	720.1–749.9 mg Mean: 734.8 mg	720.1–748.4 mg Mean: 734.3 mg
Friability	0.3%	-	-
Hardness	60–71 N Mean: 62.5 N	65–94 N Mean: 78.2 N	71–89 N Mean: 80.9 N
Disintegration time	5:35–7:00 min Mean: 6:18 min	10:00–14:00 min Mean: 12:27 min	9:30–14:10 min Mean: 11:50 min
Dissolution%	95.7–96.7% Mean: 96.1%	98.0–99.5% Mean: 98.7%	97.14–98.44% Mean: 103.7%
Assay of aloin	132 mg/tab	123.1 mg/tab	118.53 mg/tab
Content uniformity of aloin	95.9–103.5% RSD%: 2.93	96.3–103.9% RSD%: 2.92	-

**Fig. 2:**
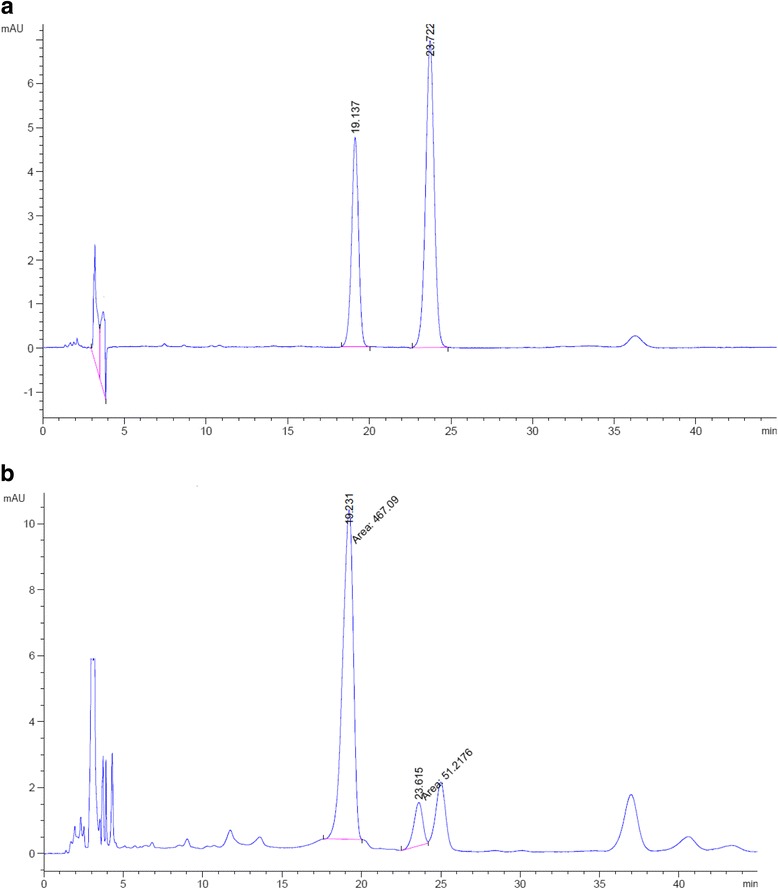
HPLC chromatogram of Aloin reference standard (**a**) and AF tablet (**b**)

The HPLC chromatograms of aloin standard solution and AF tablets have been presented in Fig. 2. The retention times (Rt) of aloin which is the sum of two diastereomers A and B were 19.1 and 23.7 min, respectively. Sum of area under the curve (AUC) of the two peaks was used for calculations. The results have been presented in Table 5.

All physicochemical characteristics of core and coated tablets were in agreement with USP requirements.

### Stability results

As shown in Table 5, no significant changes were observed in physicochemical specifications of AF coated tablets after 30 day at 40 °C temperature and 75% humidity.

### Method verification

The results of method verification have been shown in Table 6.

**Table 6:** Method verification results for determination of aloin in AF tablets

Parameter	Amount
Linear range	5–500 μg/ml
Determination coefficient (r^2^)	0.9999
Equation	y = 24.974× + 40.538
LOD	0.0053 μg/ml
LOQ	0.0161 μg/ml
Precision (Mean ± SD)	119.4 ± 1.9 mg/tab
RSD%	1.62%
Recovery%	108.0 ± 1.5%

## Discussion

There is a strong potential in the traditional and ethnomedical knowledge of various countries for developing new and efficacious drugs for the treatment of diseases [27]. Due to increasing trend for use of natural remedies among all populations, quality assurance of the herbal materials is a necessity. WHO has set specific guidelines for assessment of the safety, efficacy, and quality of herbal medicines as a prerequisite for global harmonization [2]. The oral route is the most important method of administrating drugs for systemic effects [22]. AF is an oral drug that is widely used in ITM in powder form or in a mixture with honey. In this study, coated AF tablets were prepared using different excipients for formulation. During preformulation studies, microcrystalline cellulose was applied as the diluent and disintegrant, however; the flowability of the formulation was not satisfying. Poor flow characteristics of formula may cause many problems such as non-uniformity in tablets and weight variation problems [22]. Further, starch was added as the binder, diluent and disintegrant and improved the flowability. The best formulation was achieved when the former was omitted and just the starch was used. Starch is one of the most popular materials for formulating tablets being accessible and inexpensive showing a multi role in the formulations [28]. Disintegration time is one of the most important examinations in quality control of tablets being of special importance in formulating natural products since lots of these compounds become sticky when used as powders or extracts which makes usage of disintegrates inevitable. While formulating AF, croscarmellose and crospovidon were used as disintegrant [28] but they did not improve the flowability, besides as mentioned earlier, starch in the formula also acted as the disintegrant. Four formulas were provided using the glidant, colloidal silicone dioxide, but they did not show any improvement in the formulas. In the last step, magnesium stearate as the lubricant, was used in two concentrations (0.7% and 1.4%), the latter showed better flowability for the powder. Finally, the present formulation of “*Ayaraj-e-fighara*” contains only two excipients with the expected roles. The least usage of excipients results in the lower expenses and less problems during formulation and production in industrial scale. The final prepared tablets were convex, with a green film coat and acceptable appearance. The tablets showed narrow weight variation which indicated the dosage form was acceptable and ensured that tablet contained proper amount of drug which maintained the good quality and efficacy. The tablets demonstrated good hardness and friability revealing that the tablets would not erode during transportation. The AF tablets disintegrated in less than 30 min in the disintegration test that is acceptable for herbal tablets [19]. The released aloin, as the marker of the AF tablets was more than 75% (Q) after 60 min which is in agreement with USP requirements [19]. The amount of aloin in coated tablets was determined as 123.1 mg/tab with content uniformity within 85–115%. The results obtained from the method verification according to linearity, selectivity, accuracy, precision, LOD and LOQ showed that the proposed method was suitable for the analysis of aloin in AF tablets. HPLC chromatograms of placebo, blank and sample showed that no interference with other components were present in the formulation. Also, good correlation was obtained between the standard and samples; therefore, the method was selective for aloin and the reported peaks were completely separated from the other interfering compounds. The linear relationship between the detector response and different concentrations of aloin were confirmed. The results of intra-day precision and recovery confirmed the suitability of the method for aloin quantification.

Various methods have been proposed for determination of aloin in *Aloe* spp. dried juice, extracts and products. Brown et al. determined aloin A & B in *A. vera* raw materials and finished products using HPLC method. They used C_18_ (100 × 4.6 mm, 2.6 μm) column and a gradient elution from 0.1% acetic acid in water to 0.1% acetic acid in acetonitrile at λ 357 nm. They found the method was repeatable for aloin determination in different products [29]. In another study aloin was quantitated using HPLC with C_8_ (250 × 4.6 mm, 5 μm) column and water: acetonitrile 78:22 as mobile phase in λ 220 nm [30]. Comparing to the above mentioned techniques, the current proposed method is a modified USP method for quantification of aloin which is a simple, accurate and precise technique with simple sample preparation and mobile phase in isocratic mode.

## Conclusions

AF coated tablets with acceptable physicochemical characteristics were formulated and their quality control methods were developed. In our study, USP-38 method for determination of aloin in *Aloe* spp. dried juice was used with minor modification in mobile phase for better separation. This modification helped us to establish an acceptable method for quantitation of aloin in AF tablets regarding the verification parameters. It can be concluded that this method is not only a useful tool for determining aloin in AF, but also it might be an effective quality control method for assessment of aloin in products that contain *Aloe* spp. dried juice.

## Abbreviations

AF: “Ayarij-e-Faiqra”
HPLC: High Performance Liquid Chromatography
ITM: Iranian traditional medicine
